# Supplementary figures and images for: Life-expectancy changes during the COVID-19 pandemic from 2019–2021: estimates from Japan, a country with low pandemic impact
Source: PeerJ. 2023 Aug 16;11:e15784. doi: 10.7717/peerj.15784 (PMC10439719; doi:10.7717/peerj.15784)

(A)

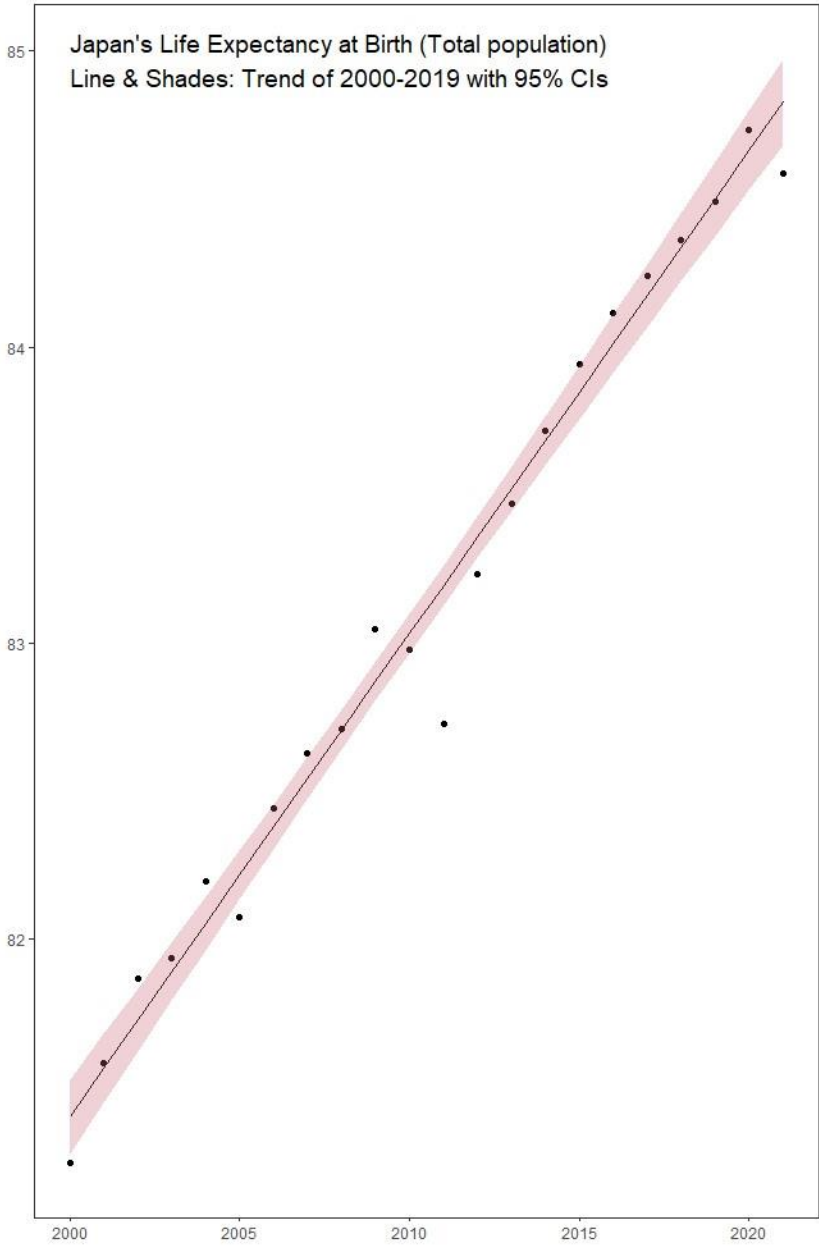

(B)

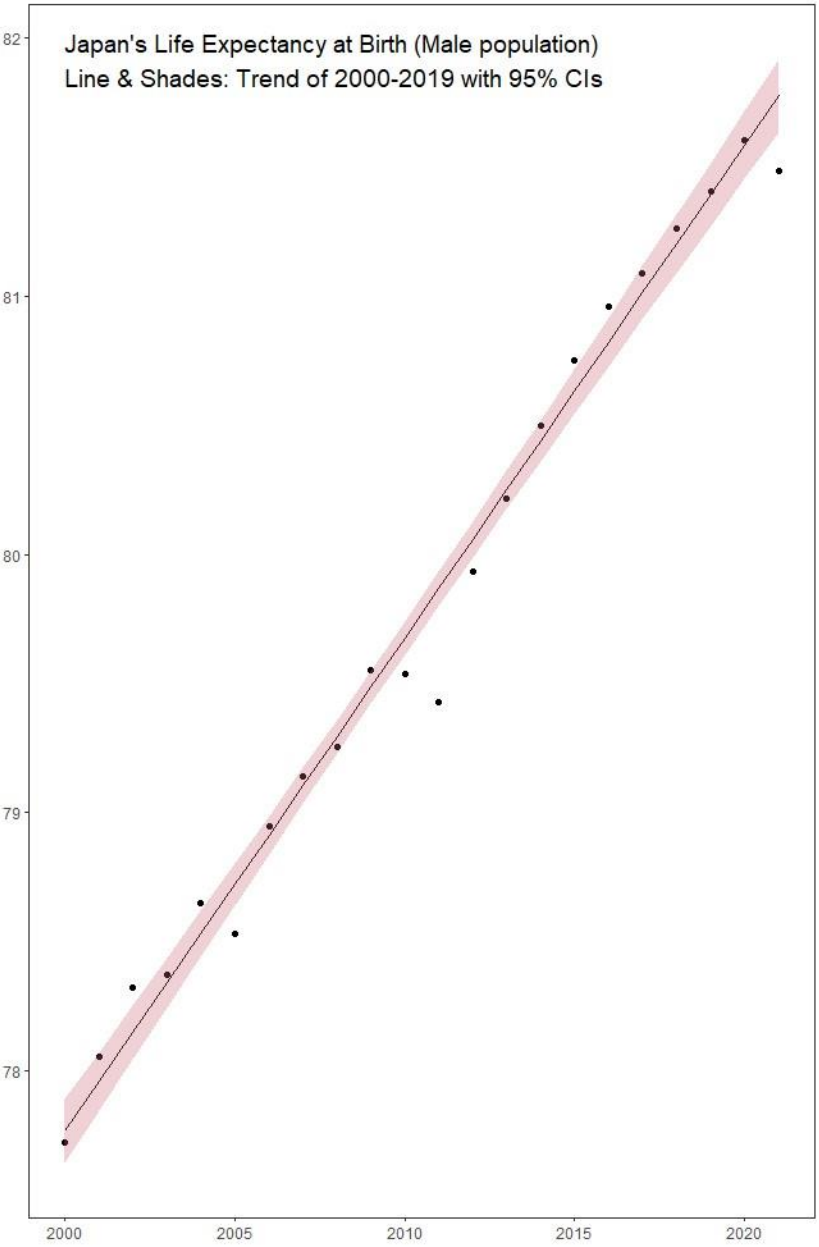

(C)

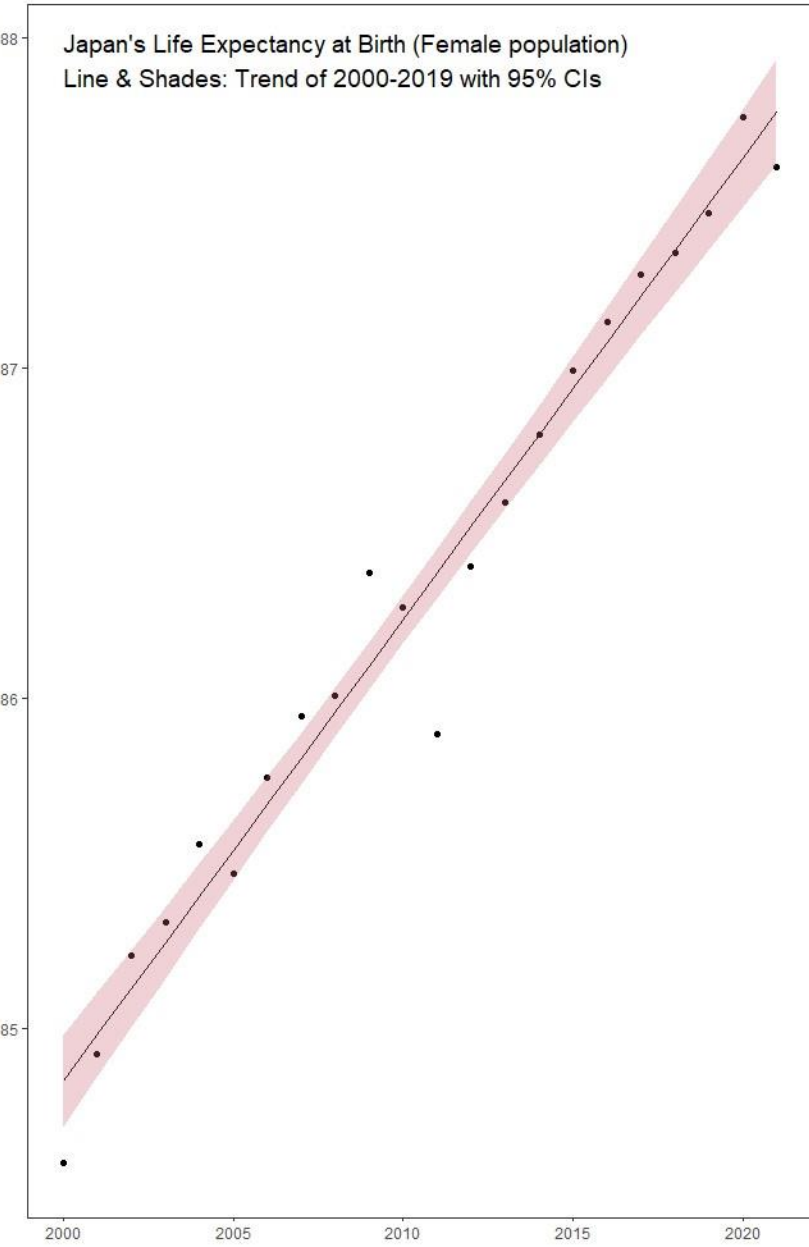

Supplement: Supplemental Information 1 — A to C: life expectancy from 2000 to 2021 in Japan for total, male, and female population are shown, respectively. In each panel, the line shows the best fit from linear regression using data from 2000 to 2019, with life expectancy as the response variable and year as the explanatory variable. For all panels, the shaded areas are 95% confidence intervals from linear regressions. [file peerj-11-15784-s001.pdf]

(A)

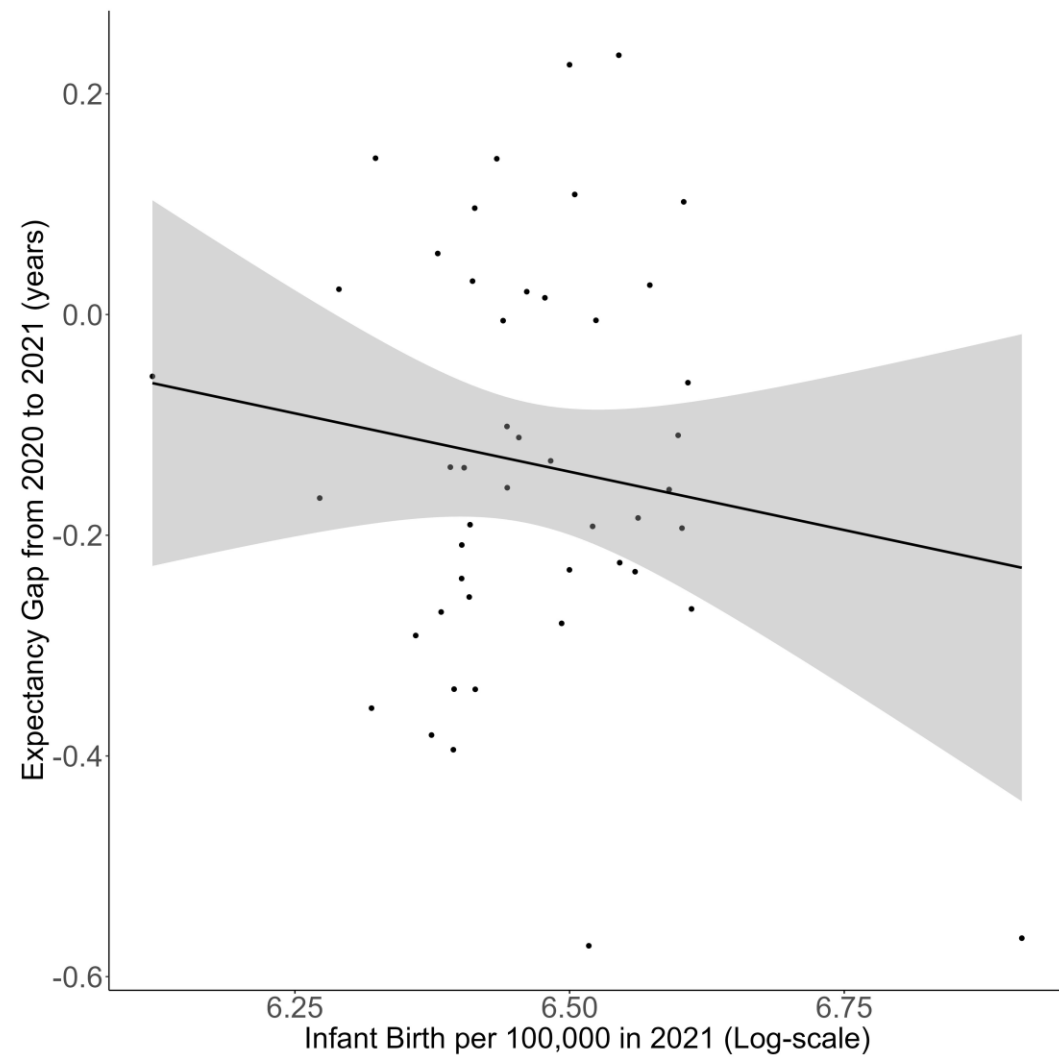

(B)

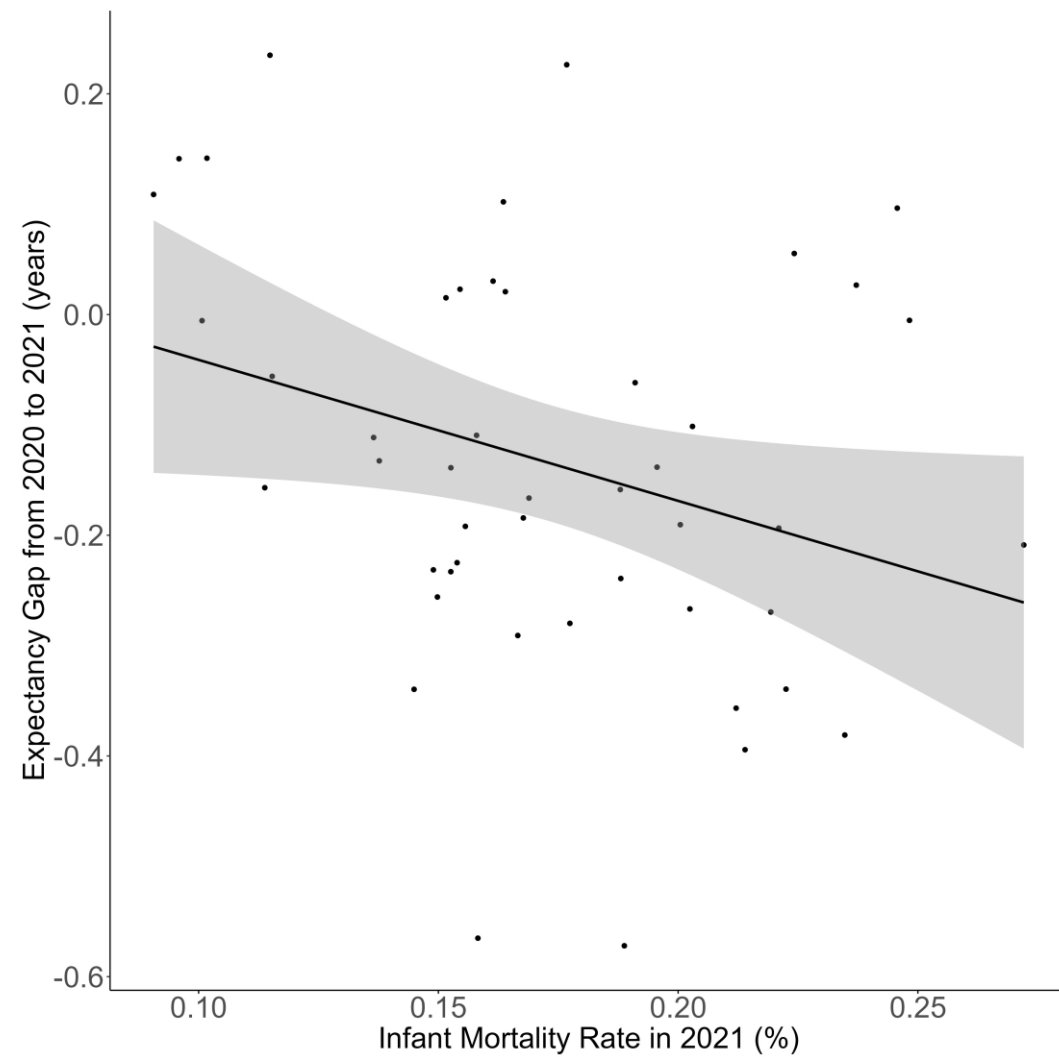

Supplement: Supplemental Information 2 — (A) Correlation between life expectancy changes in 2021 and infant birth per 100,000 (log scale) in 2021. (B) Correlation between life expectancy change in 2021 and infant mortality rate in 2021. The infant mortality rates are expressed in percentages. For each panel, the line shows the best fit from linear regression, and the shaded area is 95% confidence intervals of the fitted model. [file peerj-11-15784-s002.pdf]

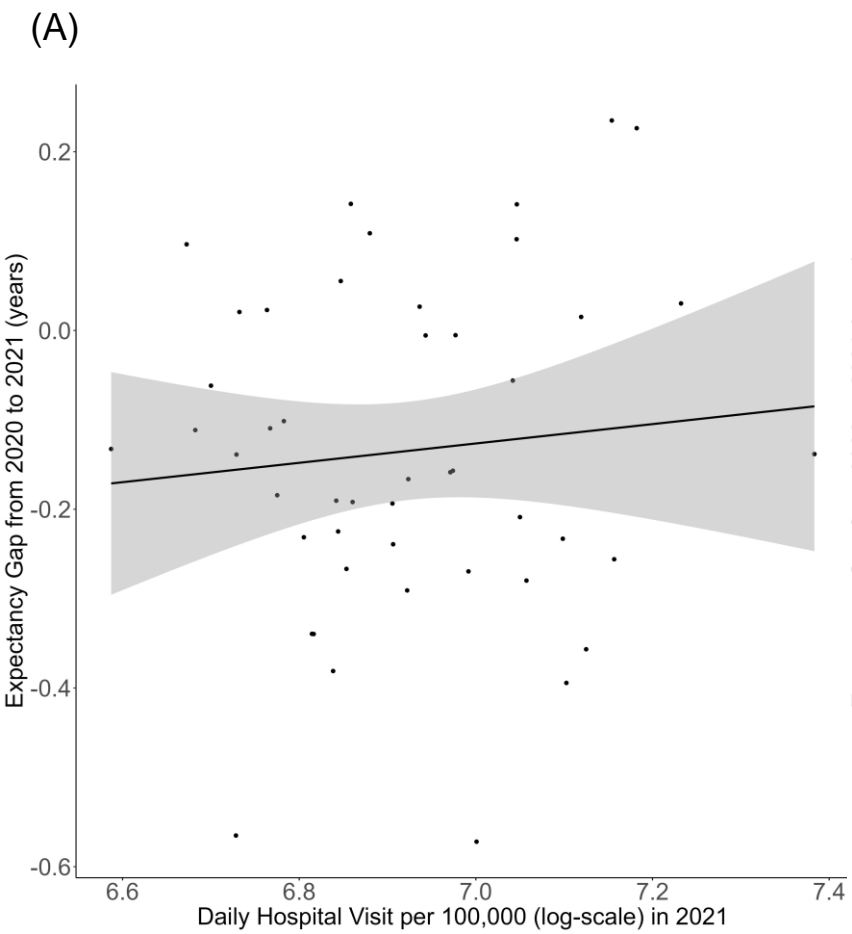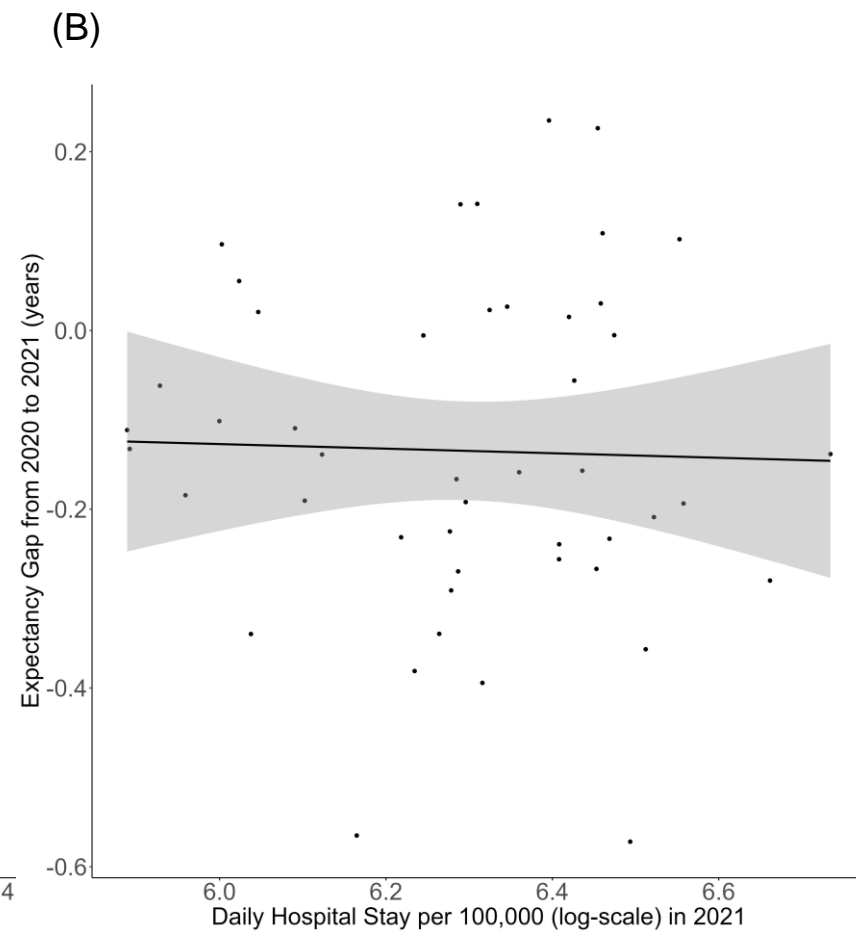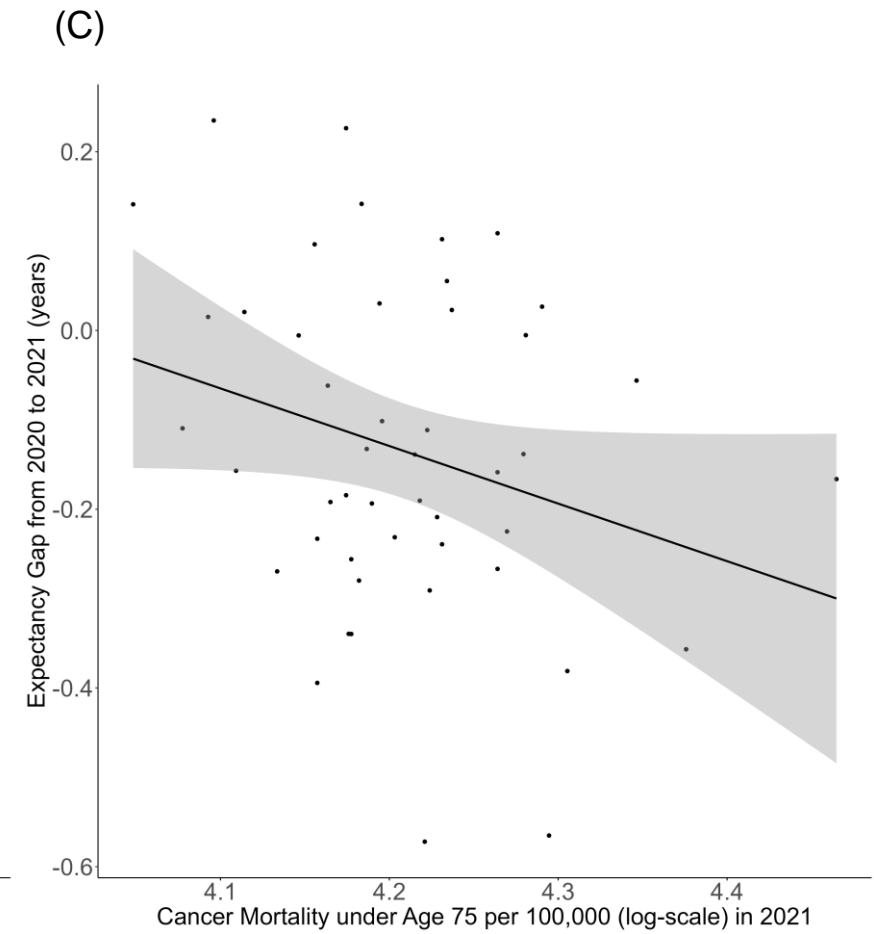

Supplement: Supplemental Information 3 — (A) Correlation between life expectancy changes in 2021 and daily hospital visit (excluding those to psychiatric hospitals) per 100,000 (log scale) in 2021. (B) Correlation between life expectancy change in 2021 and daily hospital stays in beds of general use per 100,000 (log scale) in 2021. (C) Correlation between life expectancy change in 2021 and cancer mortality under age 75 per 100,000 (log scale) in 2021. The line shows the best fit from linear regression, and the 95% confidence intervals are shown by shading. No significant correlation was found among these pairs, though cancer mortality showed mild tendency of negative correlation between life expectancy gaps. [file peerj-11-15784-s003.pdf]
